# Human Brain Atlases in Stroke Management

**DOI:** 10.1007/s12021-020-09462-y

**Published:** 2020-04-15

**Authors:** Wieslaw L. Nowinski

**Affiliations:** John Paul II Center for Virtual Anatomy and Surgical Simulation, University of Cardinal Stefan Wyszynski, Woycickiego 1/3, Block 12, room 1220, 01-938 Warsaw, Poland

**Keywords:** Human brain atlas, Stroke, Neuroimaging, MR, CT, Neuroimage processing, Diagnosis, Treatment, Prediction, Prototype, Validation

## Abstract

Stroke is a leading cause of death and a major cause of permanent disability. Its management is demanding because of variety of protocols, imaging modalities, pulse sequences, hemodynamic maps, criteria for treatment, and time constraints to promptly evaluate and treat. To cope with some of these issues, we propose novel, patented solutions in stroke management by employing multiple brain atlases for diagnosis, treatment, and prediction. Numerous and diverse CT and MRI scans are used: ARIC cohort, ischemic and hemorrhagic stroke CT cases, MRI cases with multiple pulse sequences, and 128 stroke CT patients, each with 170 variables and one year follow-up. The method employs brain atlases of anatomy, blood supply territories, and probabilistic stroke atlas. It rapidly maps an atlas to scan and provides atlas-assisted scan processing. Atlas-to-scan mapping is application-dependent and handles three types of regions of interest (ROIs): atlas-defined ROIs, atlas-quantified ROIs, and ROIs creating an atlas. An ROI is defined by atlas-guided anatomy or scan-derived pathology. The atlas defines ROI or quantifies it. A brain atlas potential has been illustrated in four atlas-assisted applications for stroke occurrence prediction and screening, rapid and automatic stroke diagnosis in emergency room, quantitative decision support in thrombolysis in ischemic stroke, and stroke outcome prediction and treatment assessment. The use of brain atlases in stroke has many potential advantages, including rapid processing, automated and robust handling, wide range of applications, and quantitative assessment. Further work is needed to enhance the developed prototypes, clinically validate proposed solutions, and introduce them to clinical practice.

## Introduction

Stroke is the most common life-threatening neurologic disorder. It is a leading cause of death and a major cause of permanent disability. Stroke has a profound effect on public health and generates huge costs for primary treatment, hospitalization, rehabilitation, and chronic care. Its management is demanding because of variety of protocols, imaging modalities, pulse sequences, hemodynamic maps, techniques and criteria for treatment, definitions of a mismatch and penumbra as well as time constraints to promptly evaluate, make decision and treat, among others (Mohr et al. 2004).

Neuroinformatics and neuroimaging play a crucial role in stroke management. In order to make diagnosis and therapeutic decision Computed Tomography (CT) and/or Magnetic Resonance (MR) imaging are used to: 1) differentiate stroke from non-strokes and stroke mimicking conditions, such as brain tumor, brain abscess or encephalitis; 2) distinguish between an ischemic and hemorrhagic stroke; 3) identify or exclude vessel occlusion; 4) identify in acute ischemic stroke the infarct (i.e., already dead tissue) and penumbra (i.e., tissue at risk of progressing to infarction, still salvageable if re-perfused); 5) identify any chronic infarct(s); and 6) identify stroke subtypes. CT scanning is usually unenhanced followed, if necessary and available, by CT angiography (CTA) and CT perfusion (CTP). Then, perfusion maps with various hemodynamics parameters can be derived from CTP including cerebral blood volume (CBV), cerebral blood flow (CBF), mean transit time (MTT), peak height (PKHT), and time to peak (TTP). Conventional and advanced MR multiple studies depict anatomy, angiography, diffusion, and perfusion. They include T1-weighted, T2-weighted, T2* gradient echo (GRE), fluid-attenuated inversion recovery (FLAIR), MR angiography (MRA), MR diffusion-weighted imaging (DWI) along with the apparent diffusion coefficient (ADC) to detect and assess the infarct, and/or MR perfusion-weighted imaging (PWI) to calculate the perfusion maps in order to delineate the penumbra.

A typical clinical practice is to process all these multiple studies individually by visual inspection. This causes the process to be time consuming in the situation when “time is brain” and not quantitative, while certain conditions have to be calculated accurately to make the therapeutic decision. In order to cope with some of these problems we have proposed a paradigm shift in stroke image processing by employing human brain atlases. This shift is from a 2D visual inspection of individual scans to atlas-assisted quantification and simultaneous visualization of multiple 2D and 3D images (Nowinski et al. 2008).

Human brain atlases are central to this approach. Over the last century brain atlases and maps have progressed tremendously from a few print cortical maps to a plethora of electronic, deformable, probabilistic, multi-modal, and multi-scale brain atlases in health and disease (Toga et al. 2006; Evans et al. 2012; Amunts et al. 2014; Nowinski 2017a). The brain atlases have been employed in a wide scope of applications ranging from medical education to research to clinics as reviewed in (Nowinski 2017a). One of clinical applications of brain atlases is neuroradiology (Nowinski 2016) and, consequently, stroke image handling. The use of human brain atlases in stroke management opens new application avenues which subject is addressed below.

I propose here some novel concepts and methods in stroke management by employing multiple human brain atlases. This novelty is confirmed by our numerous patents granted (and 17 US stroke-related patents are listed in Appendix). The way of using brain atlases in handing stroke images is also new and it has several advantages. I address a potential usefulness of and summarize our preliminary experience in the development of prototypes equipped with various brain atlases for stroke occurrence prediction and screening, rapid and automatic stroke diagnosis in emergency room (ER), quantitative decision support in thrombolysis in ischemic stroke, and stroke outcome prediction and treatment assessment.

## Methods, Materials and Applications

### Methods

The stroke applications employ three various brain atlases. These are the atlas of anatomy (Nowinski 2005), atlas of blood supply territories (Nowinski et al. 2006a), and Probabilistic Stroke Atlas (PSA) (Nowinski et al. 2014a).

The atlas of anatomy was derived from the Talairach and Tournoux (1988) brain atlas by its postprocessing, extensions, and color coding (Nowinski et al. 1997) followed by its continuous enhancements (Nowinski 2005). The atlas of blood supply territories was created to spatially correspond to the atlas of anatomy. Both atlases have the same shape of the cortex and ventricular system, share outlines of the cerebellum and brainstem, and have the same image size, number of the corresponding images, and image resolution. Consequently, these two atlases are in natural spatial registration.

The PSA combines neurological parameters with pathology localized on neuroimages for a population of stroke patients. This atlas aggregates a multiplicity of diverse parameters and presents the distribution of each parameter as a 3D image. The PSA is a special case of the probabilistic brain damage atlas for stroke lesions discussed below.

The overall method has, generally, two major steps: 1) mapping of the atlas(es) to a patient’s scan (or vice versa), and 2) atlas-assisted scan (or perfusion map) processing.

Atlas-to-scan mapping (or spatial registration) methods, producing an individualized atlas, have been reviewed in (Nowinski 2017b), and any known methods can be employed in the stroke applications discussed below. In our stroke prototypes developed we used our own atlas-to-scan mapping methods because they are very fast (of a few seconds), automatic with no parameter setting, conceptually simple (also for the clinicians to understand the underlying processing), and easier to us to modify, extend and integrate them into stroke applications. Moreover their validation, although tedious and time consuming, is conceptually straightforward. Two main methods used for atlas-to-scan mapping are the Fast Talairach Transformation (FTT) (Nowinski et al. 2006b) and an ellipse-based fitting method (Volkau et al. 2012).

The FTT is a rapid version of the Talairach transformation (Talairach and Tournoux 1988) with the modified Talairach landmarks introduced by Nowinski (2001). The original Talairach transformation subdivides the brain into 12 cuboidal regions and scales it piece-wise linearly. The FTT was implemented and its performance evaluated for MR neuroimages. The identification of the point and distributed landmarks in the scan by the FTT is fully automatic and performed in three steps: calculation of the midsagittal plane (MSP), computing of the anterior (AC) and posterior commissure (PC) point landmarks, and calculation of the six external cortical landmarks. Having the MSP and the landmarks calculated, the processed scan is reformatted in the AC-PC plane, and the atlas is scaled piecewise linearly and superimposed on the scan.

The algorithm for the MSP extraction localizes the interhemispheric fissure line segments by sampling the vicinity of this fissure with five pairs of sampling parallel line segments (that cover the white and gray matters and cerebrospinal fluid regions around the interhemispheric fissure) in a coarse and fine steps, selects fissure line segment inliers by applying a histogram-based angular and distance outlier removal, and calculates the equation of the MSP from the selected inliers by means of the least square error fit (Hu and Nowinski 2003).

The algorithm for a rapid and automatic localization of the AC and PC point landmarks combines anatomic and radiologic properties of the AC and PC and their surrounding structures (Bhanu Prakash et al. 2006). These landmarks are localized in two stages, coarse and fine. In the coarse stage the landmarks are identified on the given MSP by analyzing their relationships with the corpus callosum, fornix, and brainstem. Subsequently, in the fine stage their positions are refined in a well-defined volume of interest by analyzing locations of the lateral and third ventricles, interhemispheric fissure, and massa intermedia. The algorithm exploits simple operations including histogramming, thresholding, region growing, and 1D projections.

The modified Talairach cortical landmarks are calculated on three 2D planes, the AC-PC axial plane and two perpendicular coronal planes passing through the AC and PC landmarks, in contrast to the need of processing the entire brain in 3D as in the original Talairach transformation (Nowinski 2001). Then, for each modified cortical landmark a single coordinate is calculated, as opposed to three coordinates required for the corresponding original cortical landmark. For the calculated MSP, AC and PC, these three planes are determined, and the brain images on them are segmented based on range-constrained thresholding and morphological operations followed by local refinement to determine their extents in order to locate the cortical landmarks (Hu et al. 2005). This algorithm also exploits anatomic knowledge and employs only simple operations, like thresholding, basic morphological operations, and distance transform.

In order to increase the accuracy of the FTT without compromising its performance, we introduced two additional landmarks calculated automatically: the top of the corpus callosum and the most ventral point of the orbito-frontal cortex (Nowinski and Bhanuprakash 2005), doubling in this way the subdivision of the cerebrum from 12 to 24 cuboidal regions and increasing the number of degrees of freedom of the FTT from 13 to 15. Another extension of the FTT aimed to compensate against a variable size of the ventricular system. The standard FTT was followed by the complete and automatic extraction of the ventricular system (Xia et al. 2004) along with the determination of some point landmarks on its surface and, subsequently, by nonlinear warping based on radial functions (Nowinski et al. 2006b).

In general, the use of anatomic and radiologic domain knowledge makes the FTT component algorithms more rapid, accurate, and robust. On the other hand, these algorithms become acquisition-dependent and may require additional development. For instance, to calculate the MSP for the diffusion images and perfusion maps, we developed a dedicated algorithm based on the Kullback-Leibler’s (KL) measure quantifying the difference between two intensity distributions (Nowinski et al. 2006c). Then, the MSP is a sagittal plane with the highest KL measure.

Besides being acquisition-dependent, another limitation of the FTT (similarly to the original Talairach transformation) is that it requires the landmarks to be present in the scan. This may not be the case in the ER where typically high slice thickness CT scans are acquired. In order to overcome these limitations, while keeping the conceptual simplicity and speed of the atlas-to-scan mapping, an ellipse-based fitting method was devised (Volkau et al. 2012). The method is based on an observation that the shape of the cortex on the MSP can be well approximated by an ellipse, so practically this approach applies to any image modality as long as the cortex is visible. In addition, anatomic positions of some cortical and subcortical landmarks are related to the parameters of this elliptical approximation. The ellipse-based method applies an atlas transformation similar to that of the FTT, but the landmarks are determined differently. The method enables a statistical localization of landmarks in any, including sparse, scans, where the landmark points are hardly discernible or even absent. The method performs the following steps: calculation of the MSP by means of the KL-measure (Volkau et al. 2006a), setting a sagittal slab around the MSP and computing the maximum intensity projection (MIP) slice with the outline of the cortex (to compensate for a variable width of the interhemispheric fissure), identification of the fiducial points on the outline of the cortex on the MIP slice, fitting an ellipse to the cortex, calculation of the ellipse parameters, computation of the point landmarks, and atlas-to-scan mapping (Volkau et al. 2012). The algorithm for the calculation of the MSP based on the KL-measure works in two stages, coarse and fine (Volkau et al. 2006a). In the coarse stage the central slice in the volume along the sagittal direction is identified, a volume of interest (VOI) around this central slice in the sagittal direction is determined, the first slice of the VOI is taken as the reference slice, the KL-measure for all the slices in the VOI with respect to the reference slice is computed, and the slice with the maximum KL-measure is selected as the coarse MSP. In the fine stage, three corner points selected on the coarse MSP are perturbed and the KL-measure is iteratively calculated for a decreasing VOI. The set of the corner points giving the maximum value of the KL-measure defines the fine MSP. The outline of the cortex on the MIP slice may be set quickly interactively or any automatic skull-stripping algorithm can be used, such as (Hu et al. 2005; Sadananthan et al. 2010) for MR scans and skull-based thresholding (Puspitasari et al. 2009) for CT scans. The later approach may take longer time, be acquisition-dependent (in contrast to the whole mapping method) and create some artifacts, especially in the skull base region, whereas the fiducial points should preferably cover the entire region between the orbitofrontal and occipital cortex. The statistically determined point landmarks are ellipse-dependent; for instance, the locations of the AC and PC are simply calculated in terms of the major and minor axes of the fitted ellipse.

CT scans are infrequently acquired in the ER with a considerable head tilt, large slice thickness and high partial volume effect. In order to cope with these acquisitions, the algorithm for calculation of the MSP was extended by estimating patient’s head orientation by means of a model fitting (a skull-based fitting on the axial plane), image processing and atlas-based techniques, followed by volume reorientation for a better initialization and a subsequent MSP calculation based on the KL-measure (Puspitasari et al. 2009).

Processing of the scan with the individualized (superimposed) atlas is application-dependent. In order to process a stroke scan (or generally scans and/or maps), a combination of methods is applied including image processing, statistical modeling, atlas-assisted analysis, and population-based atlases. What is common in these applications is that the processing handles certain regions of interest (ROIs) in the scan or map. These ROIs can be two-dimensional (2D, planar) or three-dimensional (3D, volumetric). The ROIs can be determined by some characteristics of a healthy brain like anatomy or by pathology. Three situations of an ROI employment are considered here and, consequently, three types of ROIs are distinguished: atlas-defined ROIs (type I), atlas-quantified ROIs (type II), and ROIs creating an atlas (type III).

The atlas-defined ROI is a region in the scan determined by the superimposed atlas on it, such as the hippocampus, central sulcus, or Brodmann’s area 17. Then, certain operations performed over the scan can be limited only to this ROI. Subsequently, the results of operations over the atlas-defined ROIs can be further processed including their comparison or aggregation.

The atlas-quantified ROI is a particular region (typically a lesion) or regions delineated in the scan that is/are subsequently quantified by the individualized atlas or atlases. This quantification is in terms of a list of all atlas structures overlapping with this ROI, and for each structure its quantitative contribution to this ROI in terms of a volume and percentage of occupancy.

In type III ROIs (i.e., ROIs creating an atlas) the content of all the ROIs is aggregated across a patient population to form an atlas. For this purpose I introduce here a concept of a probabilistic brain damage atlas (PBDA). The PBDA is a means of aggregating data and knowledge from the previously treated patients with local brain damage. A brain damaged can result from disease or injury, such as brain tumor, stroke, trauma or infection. For each patient, the damaged (single or multi-focal) region is segmented (delineated) in the scan and some measure or parameter quantifying the brain damage or its state related to this specific region is assigned to it. The simplest way of aggregation of patient population data is by averaging, although more sophisticated aggregations taking into account the size, shape, location, and mutual overlapping of the contributing ROIs can be employed as analyzed in (Nowinski et al. 2014a). The PBDA forms a set of 3D volumes and each volume corresponding to the examined parameter is calculated as follows:

- *For each parameter*
-  *For each case/scan*
-    *Delineate ROI and create its contour file*
-    *Normalize spatially the contour file*
-    *For each voxel within the normalized contour file*
-     *Aggregate the parameter value*
-  *Divide the aggregated values by the number of cases*

The PBDA volumes can be processed, analyzed, and visualized as well as trends and knowledge extracted from them.

### Materials

Numerous and diverse cases have been used in the implemented stroke systems (prototypes) for validation. They can be arranged in four groups: 1) the ARIC (Atherosclerosis Risk in Communities) large longitudinal epidemiologic cohort study [URL, ARIC, 2020] with T1-, T2- and PD-weighted, spatially corresponding scans employed for the stroke occurrence prediction; 2) ischemic stroke and hemorrhagic stroke CT cases used for the ischemic stroke CT system and the stroke system in the ER; 3) MRI cases with multiple pulse sequences including T1-weighted, T2-weighted, FLAIR, MRA, DWI, and PWI maps (including CBV, CBF, MTT, PKHT and TTP) employed for the ischemic stroke MR system; and 4) 128 stroke cases used for the creation and validation of the PSA (selected from the cohort of 458 clinically confirmed ischemic stroke patients, chosen from a larger group of more than 700 patients), each case with CT scans (at admission and follow-up), contoured infarct(s), 170 variables (demographic, laboratory (including biochemical), and clinical measures and outcomes (including stroke scales)) gathered within one year follow-up.

### Atlas-Assisted Stroke Applications

Four stroke preliminary applications are developed and described below, where the brain atlases are critical core components, aiming to support stroke occurrence prediction, stroke detection in the ER, making treatment decision in ischemic stroke, and stroke treatment assessment and outcome prediction.

#### Atlas-Assisted Stroke Occurrence Prediction

White matter hyperintensities (WMHs) or leukoaraiosis is considered the main imaging sign of cerebral small vessel diseases. WMHs are hyperintense on FLAIR and T2-weighted sequences, and isointense or hypointense on T1-weighted sequences with respect to normal brain (Pantoni 2010). A number of studies have demonstrated that WMHs are associated with an increased stroke risk (Moran et al. 2012) and its recurrence (Kim et al. 2014). WMHs are quantified typically by the Fazekas scale (Fazekas et al. 1987) which divides the white matter in the periventricular and deep white matter, and each region is given a grade between 0 and 3 depending on the size and confluence of lesions. This grading is performed visually. Our goal was to attempt to automate this assessment and make it more quantitative and objective.

The processing for atlas-assisted stroke occurrence prediction contains the following steps:

1. Segmentation of WMHs, generally, by any method. Here, because of the nature of the available data, we employed a multi-modal segmentation of T1-, T2- and PD-weighted images with a simple, user-controlled thresholding based on the known intensity ranges (i.e., bright, iso and dark) of WMHs for these three pulse sequences.
2. Creating contours around the segmented (binary) regions with the control points for potential editing to enhance the segmented WMHs by employing the contour editor described in (Nowinski et al. 2012a).
3. Extraction of the ventricular system in 3D by an automatic, domain knowledge-driven algorithm (Xia et al. 2004). Then, the ventricular system is subdivided into multiple ROIs, and in each of them local statistics is calculated, a seed point is determined and directional region growing applied while checking anti-leakage conditions. All the unconnected regions grown are subsequently connected by relaxing the original growing conditions.
4. Quantification of the extracted binarized WMHs in terms of the number of loci, their volume, and spatial relationships with respect to the ventricular system.
5. Mapping of the anatomic atlas on the images by means of the FTT (Nowinski et al. 2006b).
6. Atlas-assisted analysis based on type II ROIs.

#### Atlas-Assisted Stroke Diagnosis in the Emergency Room

The first-line diagnosis for emergency evaluation of acute stroke is unenhanced (non-contrast) CT (Lövblad and Baird 2010). However, its sensitivity is only 25% versus 86% in MR; moreover, within the first 3 h, it is lowered to 7% for CT and 46% for MR (Chalela et al. 2007). In addition, the scans are frequently viewed initially by non-stroke clinicians (including emergency physicians, non-neuroradiologists, or even neurology or radiology residents, or junior staff on duty) before being interpreted by stroke neuroradiologists (Brown et al. 2004). Therefore, an automatic and fast detection and localization of ischemic infarcts in non-contrast CT could assist in enhancing and expediting diagnosis. Our goal is to address these issues by providing a relevant enhancing tool.

Any pathology in a brain scan typically results in asymmetry between the left and right hemispheres, so by identifying this asymmetry, a pathology in the scan can be detected (Volkau et al. 2006b). This global comparison of the hemispheres can be followed by a more specific local comparison for a set of numerous pairs of ROIs placed on both hemispheres, and those ROIs can be generated by a brain atlas. The developed atlas-assisted stroke system in the ER exploits two atlases to define such pairs of ROIs: atlases of anatomy and atlas of blood supply territories. The system supports rapid and automatic stroke detection, distinguishes between an ischemic and hemorrhagic stroke, and localizes an infarct or hemorrhage. It analyzes statistically the differences between the left and right hemispheres in multiple ROIs delineated by the brain atlases of anatomy and blood supply territories. The comparison between the left and right hemispheres is performed in 3D within the corresponding atlas ROIs rather than in acquired images, avoiding image asymmetry resulting from a potential head tilt.

The processing steps for atlas-assisted stroke diagnosis in the ER exploit fast and dedicated algorithms and are as follows:

1. Calculation of the midsagittal plane (Volkau et al. 2006a; Puspitasari et al. 2009).
2. Extraction of the ventricular system to determine cerebrospinal fluid regions. Three algorithms have been developed for this purpose, 1) the abovementioned algorithm for extraction of the ventricular system from MR scans (Xia et al. 2004), 2) an algorithm for extraction of the ventricular system from CT scans (Liu et al. 2010), and 3) a dedicated algorithm for ventricular system extraction from ischemic stroke CT scans (Poh et al. 2012). The algorithm by Liu et al. (2010) is model-guided and employs domain knowledge about the anatomy, shape variation and intensity distribution of the normal ventricular system in CT neuroimages. A 3D model of the ventricles derived from the anatomy atlas is first registered to the scan linearly against the brain’s bounding box. The registered model defines multiple regions in the ventricular system. Then, in each region thresholds are calculated and the cerebrospinal fluid segmented. The automated algorithm by Poh et al. (2012) is template-based and employs two ventricular templates: one is for the normal brain and the other is pathologic built from several brains with substantially enlarged ventricles. The templates are registered piece-wise linearly to the scan by employing the ellipse-fitting method (Volkau et al. 2012) with the MSP calculation algorithm by Puspitasari et al. (2009) to accommodate for large head tilts. An ROI is determined by means of the pathologic template, which limits potential “leakage” of the cerebrospinal fluid region into the ischemic region, as their densities overlap. A suitable threshold is computed taking into account the distributions of cerebrospinal fluid, white matter and gray matter calculated by means of the algorithm by Gupta et al. (2010), and a thresholding is performed in the ROI, followed by artifact removal.
3. Skull removal and brain extraction by performing skull-based thresholding (Puspitasari et al. 2009).
4. Removal of the cerebrospinal fluid regions from the extracted brain because the cerebrospinal fluid density range overlaps with that of infarcts.
5. Rapid atlas to CT scan mapping using the ellipse-fitting method (Volkau et al. 2012).
6. Atlas-assisted analysis based on type I ROIs by comparison of all the corresponding left and right ROIs for both atlases, and a diagnosis is made based on testing of multiple statistical conditions (like a mismatch between the ROIs density distribution curves).
7. Producing a report with the diagnosis (infarct, hemorrhage, infarct with hemorrhagic transformation, or not detected) and lesion location details (CT slices, anatomic structures and blood supply territories).

#### Atlas-Assisted Treatment Decision Making in Ischemic Stroke

Thrombolysis is the main treatment for acute ischemic stroke delivered by administering tissue plasminogen activator (t-PA) intravenously and/or intra-arterially through a microcatheter (Mohr et al. 2004). Intravenous (IV) thrombolysis is the most direct treatment of most ischemic strokes. Uncontrolled thrombolysis may result in hemorrhagic transformation, so the procedure is applied provided that certain thrombolysis conditions are met. Depending on a protocol employed, three conditions are checked: 1) the size of the infarct to that of the middle cerebral artery (MCA) territory, 2) the presence of a diffusion-perfusion mismatch measured as the perfusion lesion to the diffusion lesion (infarct) ratio, and 3) the site of vessel occlusion, if any. The ratio of an infarct volume to that of the MCA territory predicts hemorrhagic transformation when greater than, depending of various authors, one half (Parsons and Davis 2006) or one third (Hacke et al. 2004). It should be noted that these conditions do not take into account locations of the infarct and penumbra. Our goal is to automate the calculation of the thrombolytic conditions and to extend the procedure by taking into account infarct and penumbra locations.

The atlases of anatomy and blood supply territories are employed for automatic and quantitative assessment of the infarct and penumbra taken as ROIs and, additionally, to calculate the ratio of the infarct volume to that of the MCA territory in 3D in contrast to standard procedure of assessing it on some (usually one or two) selected slice(s). An atlas-assisted analysis provides the complete list of anatomic structures and blood supply territories, along with their volume and percentage of contributions to the infarct and penumbra regions.

The processing for atlas-assisted treatment decision making in ischemic stroke is the following:

1. Calculation of the midsagittal plane by means of various algorithms depending on the pulse sequence (Hu and Nowinski 2003; Nowinski et al. 2006c; Volkau et al. 2006a).
2. Rapid atlas to scan mapping by means of the FTT for MR scans (Nowinski et al. 2006b) or by the ellipse-based method for CT scans (Volkau et al. 2012).
3. Infarct and penumbra (ROIs) segmentation. The infarct is segmented automatically by means of a divergence measure taken here as a ratio of the intensity probability density functions for the left and right hemispheres (Bhanu Prakash et al. 2008). The penumbra is delineated interactively by employing a dedicated contour editor (Nowinski et al. 2012a). These operations are followed by the calculation of the infarct and penumbra volumes and their ratio.
4. Atlas-assisted analysis based on type II ROIs and decision making support (Nowinski et al. 2006a).

#### Atlas-Assisted Treatment Assessment and Outcome Prediction

For an atlas-assisted treatment assessment and outcome prediction, the probabilistic stroke atlas (PSA) is employed. We have created the PSA and studied its properties for ischemic stroke (Nowinski et al. 2014a). The PSA is a special case of the PBDA. Then, a set of PSA volumes has been constructed taking non-contrast CT as a scan, an ischemic lesion (infarct) delineated on the scan taken as an ROI (of type III), and numerous parameters (out of 170 acquired) assigned for ROI quantification, including the modified Ranking Scale and the Barthel Index at day 7, 30, 90, 180 and 360.

For any studied parameter, the patient’s CT scan is superimposed on its corresponding PSA volume, the infarct is delineated on the scan forming the ROI in 3D, and the predicted values of this parameter are read from the PSA within the ROI.

The processing steps for atlas-assisted treatment assessment and outcome prediction are the following:

1. Delineate an infarct in the scan forming the ROI in 3D; it was done rapidly interactively by using a dedicated contour editor (Nowinski et al. 2012a).
2. Select a suitable PSA map or maps with the parameter(s) to be predicted.
3. Map the patient’s scan to the PSA map(s) by means of the ellipse-based method (Volkau et al. 2012); additionally, the anatomic atlas can also be superimposed on the scan to provide anatomic localization.
4. Read the prediction (parameter values) in the ROI from the selected PSA maps (Nowinski et al. 2014a).

It shall be noted that type III ROIs are employed for atlas creation, whereas type II ROIs for atlas use in prediction.

## Results

The stroke applications presented below this is work in progress and some preliminary results are given. All four stroke applications were implemented as working prototypes (proofs of concept) resulting from integration of multiple methods and brain atlases. These prototypes were presented to both neuroradiological and neurological communities. All four prototypes were demonstrated as education exhibits at RSNA (Radiological Society of North America) annual meetings receiving four awards. The prototypes were also demonstrated at the European Congress of Radiology, World Congress of Neurology, and American Society of Neuroradiology. Moreover, some of them have been trial licensed globally to numerous industrial (including Siemens and Philips) as well as clinical and research institutions in USA, Canada, France, Germany, Poland, Italy, Belgium, Singapore, India, and Australia. In addition, our stroke-related work was nominated for Asian Innovation Award as well as resulted in numerous patents granted (see Appendix) and pending.

The prototype for the atlas-assisted stroke occurrence prediction employs the FTT for mapping of the anatomic atlas to T1-, T2- and PD-weighted scans. The component algorithms for atlas-to-scan mapping, including the calculation of the MSP, AC and PC point landmarks, and the external cortical landmarks, were validated first followed by the validation of the entire FTT.

The algorithm for the localization of the AC and PC point landmarks was initially validated on 94 diversified datasets resulting in the mean distance errors of 1.02 mm for the AC and 0.97 mm for the PC (Bhanu Prakash et al. 2006). The fine stage processing doubles the accuracy. The validation also revealed some limitations of the algorithm, namely, it is not applicable for modalities unable to clearly delineate the corpus callosum, fornix and/or brainstem, it is not able to localize the AC and PC when the MSP is rotated for more than 35°, and the results may be incorrect for the slice thickness greater than 3.5 mm and the pixel size larger than 2 × 2 mm (taking into account a small size of the AC and PC structures).

The algorithm for the extraction of the MSP was initially validated on 125 MR and CT normal and pathological diverse cases (from 10 centers in 3 continents). In addition, its robustness to noise, asymmetry, rotation, bias field and sensitivity to parameters was studied. The algorithm extracts the MSP with the average angular and distance errors of (0.40°, 0.63 mm) for normal and (0.59°, 0.73 mm) for pathological cases (Hu and Nowinski 2003).

The algorithm for the extraction of the modified Talairach cortical landmarks was validated on 62 diversified MR scans resulting in the average landmark location errors below 0.9 mm (Hu et al. 2005).

The algorithm for extraction of the ventricular system was validated qualitatively on 68 and quantitatively on 38 MRI normal and pathological cases. It runs successfully for normal and pathological cases provided that the slice thickness is lower than 3.0 mm in axial and 2.0 mm in coronal directions, and there is no high inter-slice intensity variability. The algorithm also works satisfactorily in the presence of up to 9% noise and up to 40% image inhomogeneity. The mean overlap metric between the results of a radiology expert and the algorithm was 0.97 (Xia et al. 2004). The extraction time is below 6 s.

Subsequently, these components algorithms were integrated within the FFT platform and the accuracy of all the landmarks was assessed on a bigger set of 215 diversified MR cases. For each case the ground truth was set in terms the interhemispheric fissure and all eight point landmarks. The average distance errors in point landmark localization were (in mm): 1.16 (AC), 1.49 (PC), 0.08 (cortical left), 0.13 (cortical right), 0.48 (cortical anterior), 0.16 (cortical posterior), 0.35 (cortical superior), and 0.52 (cortical inferior) (Nowinski et al. 2006b). The FTT calculations took about 5 s. When radial functions-based nonlinear warping was included along with the calculation of the ventricular system, this time increased a few times depending on the number of the ventricular landmarks. Quality-wise, when excluding certain distortion artifacts that occurred, generally the atlas-to-scan correspondence for subcortical structures was improved, but its quantitative anatomic validation for patient-specific scans is not straightforward.

These methods proposed for the atlas-assisted stroke occurrence prediction are novel, as confirmed by the US patents granted (listed in the Appendix); namely, Patent 3 (AC and PC calculation), Patent 6 (external cortical landmarks extraction), Patent 7 (MSP calculation), Patent 12 (the FTT), and Patent 14 (ventricular system extraction).

**Fig. 1:**
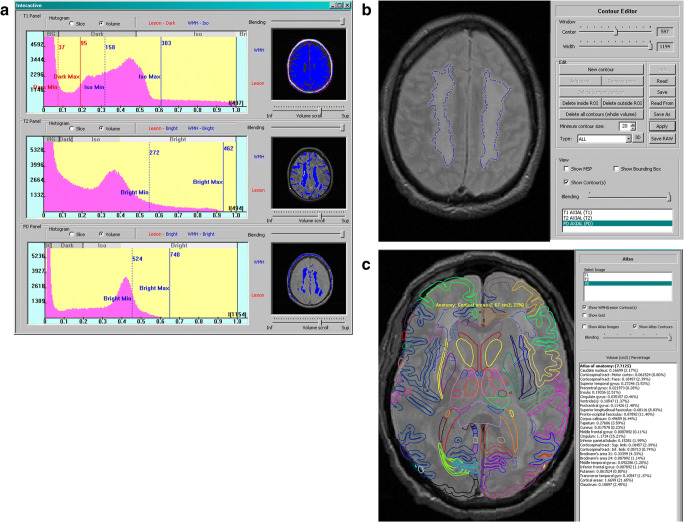
Atlas-assisted stroke occurrence prediction: a) histograms (on the left) of the T1- (top), T2- (middle), and PD-weighted (bottom) images of a selected axial plane shown along with the marked lower and upper thresholds (as the vertical lines on the histograms) and the resulting corresponding accumulated segmented WMHs regions (on the right); b) WMHs contoured on an PD axial image along with the control points enabling contour editing by the user by the provided Contour Editor; c) atlas-assisted analysis containing the individualized anatomic brain atlas in a contour format (to avoid blocking of the scan by the atlas colored regions) superimposed on a PD axial image with the segmented WMHs along with the list of structures, and for each structure its volume and percentage contributing to the WMHs is calculated (in this way the atlas provides quantification of the WMHs). Note the availability of numerous functions for image manipulation, display and result editing

All the component algorithms and the prototype of the atlas-assisted stroke occurrence prediction system were implemented in C++. The prototype was preliminarily evaluated on a few cases from the ARIC cohort study, and some resulting images along with a few windows of the user interface of the prototype are illustrated in Fig. 1. This figure shows T1-, T2- and PD-weighted images along with their corresponding histograms on which the thresholds are marked; the process of multi-modal extraction of the WMHs; the extracted WMHs in a contour form with the control point for potential interactive enhancement; and an individualized anatomic atlas with atlas-assisted analysis. This analysis provides the list of structures and for each structure its volume and percentage contributing to the WMHs is calculated (in this way the anatomic brain atlas provides quantification of the WMHs). The prototype is also equipped with numerous functions for image manipulation, display and result editing.

This application is able to provide a wealth of information. It quantifies directly the WMHs themselves (in terms of size and numbers) and provides atlas-enabled quantitative assessment of the complete underlying anatomy involved in the WMHs. As the ventricular system and the WMHs are both extracted in 3D, their spatial relationship can also be easily calculated.

The prototype for the atlas-assisted stroke diagnosis in the ER employs the ellipse fitting method with the statistical calculation of the landmarks for mapping of the mutually co-registered atlases of anatomy and blood supply territories to a scan. The ellipse-based method exploits the MSP calculated by means of the KL-measure (MSP-KL). The algorithm for MSP-KL calculation was evaluated on 75 MRI cases, additional CT and MRA datasets, and the phantom data to study its robustness against rotation, inhomogeneity and noise. The average distance and angular deviation from the ground truth were 1.25 pixels and 0.63°; moreover, the algorithm works for various modalities, pulse sequences and copes with pathological cases, and its processing time is less than 5 s for a typical dataset (Volkau et al. 2006a). The robustness of the algorithm to rotation for the phantom data was from −15° to 15° in each of yaw, pitch and roll directions, and from −7° to 7° in roll direction combined with any other. As this robustness was insufficient for handling ER acquisitions, the algorithm was subsequently extended to cope with a high head tilt by performing volume pre-rotation before MSP-KL calculation.

The extended algorithm was validated on 208 clinical scans acquired mostly in the ER with a substantial head tilt and the slice thickness ranging from 1.5 to 6 mm (with 95% cases having the slice thickness of 5 mm or more). The mean distance and angular errors (the discrepancies between the calculated MSP and the ground truth MSP) were 0.15 mm and 0.03°, and the maximum errors were 2.58 mm and 3.45° (Puspitasari et al. 2009). When tested against data rotation, the algorithm was able to successfully extract the MSP up to 40° in yaw, 30° in pitch, and 25° in roll directions, with the mean distance and angular errors of 0.64 mm and 0.71°. The algorithm takes in average 10 s to process a typical CT case.

The formulae for statistical landmark localization were determined based on analysis of 53 structural scans without detectable pathology, yielding the mean localization errors (in mm) of 2.57 for the AC and 2.69 for the PC (Volkau et al. 2012). These (AC;PC) errors are inter-slice gap dependent amounting (0.82;1.41) for gap = 0 mm and (2.93;2.06) for gap = 7 mm. Ellipse-based registration was studied for several multi-modal (CT, MR, PET) scans and hemorrhagic stroke time-series CT, and the process takes less than 15 s.

The algorithm for extraction of the ventricular system from CT scans was tested on 50 stroke patients from various sites achieving an average overlap between the extracted and ground truth ventricles of 85% in about 10 s (Liu et al. 2010).

The performance of this standard algorithm seemed sufficient in speed and accuracy. However, the accuracy of removal of the cerebrospinal fluid from the ischemic stroke images impacts stroke detection, and therefore a new dedicated algorithm was developed and a more thorough testing carried out. The algorithm dedicated for extraction of the ventricular system from ischemic stroke CT scans by Poh et al. (2012) was tested on 102 ischemic stroke scans of a slice thickness variable between 3 and 6 mm, and significant variations in the image quality, orientation, size and shape of the ventricles. More than 80% of the CT scans had visible ischemic infarcts of various size, shape and location, with their density overlapping or close to that of the cerebrospinal fluid. The sensitivity and the Dice similarity coefficients of the algorithm were 0.74 and 0.80, compared to those by Liu et al. (2010) of 0.83 and 0.69. The calculations took less than 30 s. Thus, the dedicated algorithm runs 3 times longer than that by Liu et al. (2010) and both have a comparable sensitivity and dice similarity coefficient. However, other metrics show a substantial advantage of the stroke dedicated algorithm by Poh et al. (2012) over the standard algorithm by Liu et al. (2010). Namely, conformity was 0.45 for the dedicated algorithm versus −3.09 for the standard algorithm, where a negative value in conformity means that the total number of erroneous voxels is greater than the total number of correctly segmented voxels. Sensibility was 0.88 for the dedicated algorithm versus −2.40 for the standard algorithm, where a negative value in sensibility means that the total number of over segmented voxels is greater than the total number of voxels in the ground truth.

These methods proposed for the atlas-assisted stroke diagnosis in the ER are novel, as confirmed by the US patents granted; namely Patent 2 (MSP-KL calculation), Patent 4 (image density distribution calculation), Patents 9, 11, 15 (related to pathology detection), Patent 16 (statistical landmark localization), and Patent 17 (ellipse-based registration).

**Fig. 2:**
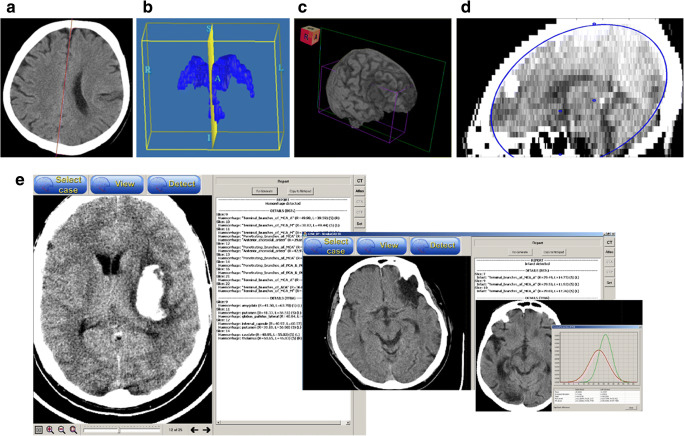
Atlas-assisted stroke diagnosis in the emergency room: a) midsagittal line extracted on a sample axial unenhanced CT image; b) ventricular system segmented in 3D along with the midsagittal plane; c) extracted brain in 3D that is volume rendered with a clearly discernible ischemic infarct; d) atlas to CT scan mapping by means of the ellipse-fitting method (the positions of the anterior and posterior commissures are also marked on this scan having a high slice thickness); and e) results of atlas-assisted analysis: left) hemorrhage detection (an axial image with a hemorrhage is shown on the left with the report for the whole brain on the right containing the details regarding the numbers of the hemorrhagic slices, affected blood supply territories (top) and anatomic structures (bottom) along with the corresponding intensity values (scaled to 8 bits) for the right and left hemispheres), center) infarct detection (an image with a part of the report), and right) left-right ROI intensity distribution curves with an axial image showing the infarcted areas (note a substantial mismatch in the corresponding curves in terms of their height, mean, and standard deviation)

All the component algorithms and the prototype of the atlas-assisted stroke diagnosis in the ER system were implemented in C++. This prototype was preliminarily evaluated on a several ischemic and hemorrhagic stroke cases providing for each case a correct decision. Several resulting images and the user interface along with some windows of the prototype are presented in Fig. 2. It illustrates the results of the component operations including the midsagittal plane extraction, segmentation of the ventricular system, brain extraction with a volume rendered ischemic infarct, and atlas-to-scan mapping by the ellipse-fitting method. The prototype works in three modes selectable from the top bar of the user interface: 1) *Select case* to enable the user to choose a scan for detection, 2) *View* to show the selected scan and scroll images, and 3) *Detect* to run the scan detection and display the report on atlas-assisted analysis with diagnosis. The generated report contains two optional parts: Infarct detected and Hemorrhage detected. Each part has two sections, one with the analysis of the blood supply territories atlas (BSTs) and the other with the analysis of the atlas of anatomy (TTBA). For any left-right hemisphere mismatch, information from all slices involved is displayed and for every slice its number is shown, the list of names of the blood supply territories or anatomic structures involved is given, and for every name the averaged values of image intensity (scaled to 8 bits) in the corresponding ROIs in the right and left hemispheres are provided. In addition, the user can display the left-right ROI mismatch as a pair of intensity distribution curves and is able to set the ROI values that discriminate between normality and pathology by setting the differences between the mean values, standard deviations, and peak heights.

The prototype of the atlas-assisted treatment decision making in ischemic stroke system employs the atlas-to-scan mapping algorithms whose validation was addressed above. The novel component algorithms are for an automatic infarct segmentation (Bhanu Prakash et al. 2008) and for a calculation of the MSP in diffusion and perfusion images (Nowinski et al. 2006c).

The algorithm for infarct segmentation was validated on 57 DWI volumes with intra 5–14 mm plane resolutions. Its median sensitivity, specificity, and Dice similarity coefficient were 86.34%, 99.83%, and 0.72, respectively (Bhanu Prakash et al. 2008). The algorithm takes 3–5 s per volume.

The algorithm for calculation of the MSP in diffusion and perfusion images was validated quantitatively on 11 DWI scans, and 50 (10 × 5) perfusion maps including CBF, CBV, MTT, PKHT, and TTP acquired for 11 stroke patients with both small and large ischemic lesions. The average angular errors were less than 1° for DWI and less than 2° for CBF and CBV; the average distance errors measured in the worst case (i.e., on the brain’s bounding box) were less than 2.5 mm for DWI and less than 5 mm for CBF and CBV (Nowinski et al. 2006c). The results obtained for the other perfusions maps (MTT, PKHT, TTP) were inferior; hence, processing of CBF or CBV is preferred for an accurate and robust calculation of the MSP. The calculation of the MSP takes about half a second.

The same stroke cases with diffusion and perfusion images were used for testing of the whole system. For all of them, the thrombolysis conditions were successfully calculated including the perfusion to diffusion lesion ratio and the size of the infarct to that of the middle cerebral artery territory (and additionally, to those of the anterior and posterior cerebral arteries). The third condition of thrombolysis regarding the vessel occlusion was initially attempted to be checked by performing vessel segmentation, 3D modeling and their subsequent surface rending, but this approach was too time consuming (an alternative solution was presented in Patent 9). Finally, the infarct and penumbra regions were quantified by means of the both atlases.

These methods proposed for the atlas-assisted treatment decision making in ischemic stroke are novel, as confirmed by the US patents granted; namely those patents related to atlas-scan-mapping mentioned above, and additionally Patent 1 (infarct segmentation) and Patents 10 and 13 (specific to atlas-based processing of diffusion and perfusion stroke images).

**Fig. 3:**
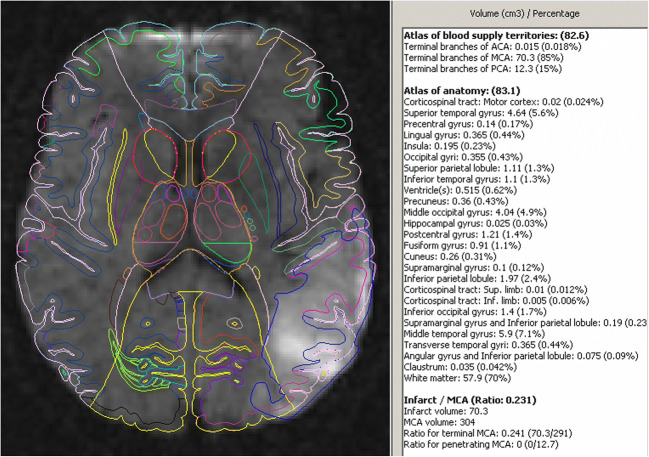
Atlas-assisted treatment decision making in ischemic stroke: left) a DWI axial image with a delineated infarct (in blue) and the individualized anatomic and blood supply territories atlases in contour representation superimposed on the image; right) atlas-assisted analysis with a contribution of the blood supply territories (top) and anatomic structures (middle) to the infarct in terms of name, volume in cm^3^ and percentage as well as the assessment of the ratio of the infarct to the whole middle cerebral artery (MCA) territory including that for the terminal and penetrating branches (bottom)

All the component algorithms and the prototype of the atlas-assisted treatment decision making in ischemic stroke system were implemented in C++. This prototype is illustrated in Fig. 3 showing the individualized anatomic and blood supply territories atlases superimposed on a DWI image with a delineated infarct as well as an atlas-assisted analysis with a contribution of the anatomic structures and blood supply territories to the infarct in terms of name, volume and percentage. Moreover, the assessment of the ratio of the infarct to the middle cerebral artery (MCA) territory is calculated for whole MCA and its terminal and penetrating branches.

The prototype of the atlas-assisted treatment assessment and outcome prediction system employs scan normalization algorithms whose validation was addressed above. The following major PSA maps were created: modified Ranking Scale (mRS) maps at day 7, 30, 90, 180 and 360, Barthel Index (BI) maps at day 7, 30, 90, 180 and 360, NIH Stroke Scale (NIHSS) at admission, NIHSS at day 7, Stroke (infarct) frequency map, Age map, White blood cell count map, Glucose ER map, and C reactive protein map. Note that except the infarct frequency maps, all the other maps were created from textual parameters.

Prediction properties of the PSA were studied in terms of data aggregation and data selection. The PSA was examined for eight variants of different data aggregation schemes and three selection variables (infarct volume, NIHSS at admission, and NIHSS at day 7), with each variable in four ranges resulting in (8x4x4x4) 512 instances. The outcome was predicted for 9 parameters (mRS at day 7, and mRS and BI at day 30, 90, 180, and 360). Therefore, to predict all 128 cases, 589,824 PSA instances were calculated. The prediction was done using a leave-one-out approach, meaning that each patient was predicted based on the PSA constructed from the remaining 127 patients (i.e., excluding the predicted patient). Hence in total, during these analyses the cases were processed 74,907,648 times. Then, for instance, the average prediction error (the absolute difference between the predicted and actual values) for mRS in the grade range of [2–5] was around one grade, i.e., 1.096 ± 0.564. In general, the PSA constrained by two parameters (the infarct volume and NIHSS at admission) reduced the average prediction error by a fraction of 0.796; the use of three patient-specific parameters further lowered it by 0.538 (Nowinski et al. 2014a). Prediction takes about 8.5 s per patients.

The method used for scan normalization employed in the PSA construction and PSA-based prediction are novel and they are patented as already mentioned above. In addition, patent applications US (20,120,246,181 A1) and EU (EP2504781 A4) have been filed on the construction and use of the PFA for diagnosis and prediction.

**Fig. 4:**
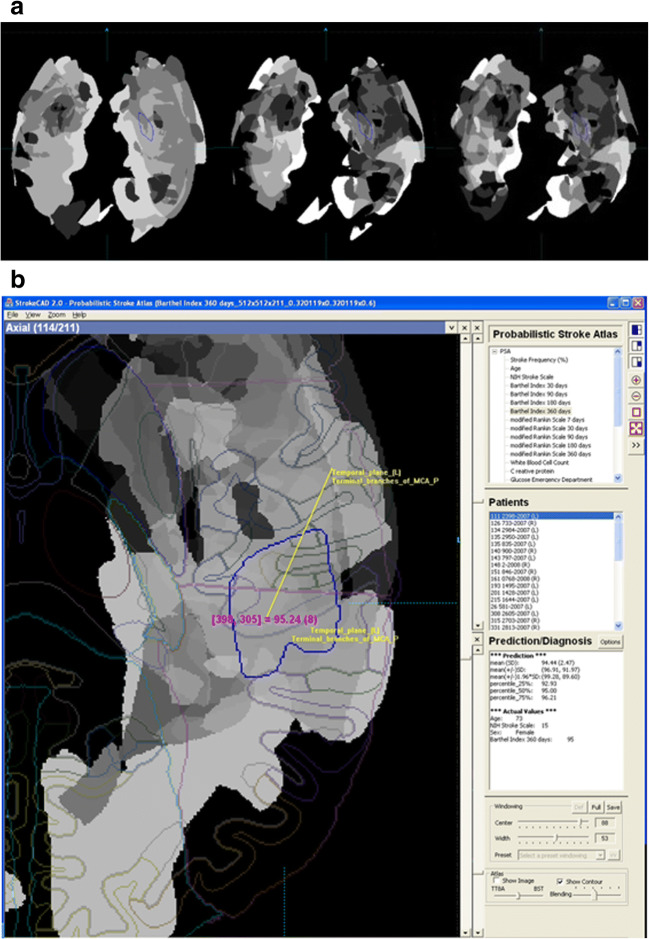
Atlas-assisted treatment assessment and outcome prediction: a) examples of PSA mRS (modified Rankin Scale) axial maps, all at the same level (shown in the radiologic convention) at day 7 (left), 180 (middle) and 360 (right); note the trends over time at the same locations demonstrating the decrease in intensity corresponding to the improvement of outcomes over time (due to the patients with longer survival); b) user interface of the system with a patient infarct contour in blue (the examined ROI) superimposed on a PSA BI (Barthel Index) at day 360 axial map along with the anatomic atlas (left) and the prediction results read in the ROI with the mean, standard deviation, and 25%, 50% (BI of 95) and 75% percentile values along with the actual value of BI of 95 for this patient (bottom-right). Note that the atlas of anatomy facilitates anatomic localization of the infarct on the PSA map. Top-right: the selectable list of the PSA maps; center-right: the selectable list of examined patients

The prototype of the atlas-assisted treatment assessment and outcome prediction system was developed in C++. The user interface of this prototype along with some results are illustrated in Fig. 4. It shows three sample axial PSA mRS maps at the same level corresponding to day 7, 180 and 360, as well as a PSA-based prediction. For prediction, a suitable PSA volume is selected from the list (in this case the Barthel Index at day 360) followed by the selection of the patient’s ID. The selected PSA map along with the normalized delineated ischemic infarct, and the outlines of the anatomic atlas are displayed. The calculated prediction value of the selected parameter along with its actual value (measured in the one-year time followed-up neurologic assessment) are both given for comparison. In addition, the user interface provides functions for data selection, image manipulation, and display.

## Discussion

I have proposed novel concepts and methods in stroke handling assisted by human brain atlases. Their novelty has also been confirmed by numerous patents granted. The proposed solutions are discussed here mainly from a brain atlas perspective in order to create an awareness and present a still unexploited potential of human brain atlases in stroke management.

We have employed multiple human brain atlases to handle various problems in stroke and, to our best knowledge, our work is the first use of human brain atlases in stroke management. In addition, this kind of atlas use is novel, going beyond a standard atlas-aided segmentation and labeling of neuroimages. Atlas-assisted stroke image processing is ROI-related and three types of ROIs are distinguished: atlas-defined ROIs (type I), atlas-quantified ROIs (type II), and ROIs creating an atlas (type III). An ROI can be defined either by atlas-guided anatomy (including blood supply territories) or scan-derived pathology. The atlas can be applied either to define ROI or to quantify it.

There are only a few works on human brain atlases in stroke. A stroke atlas as a collection of unregistered stroke images was developed by Liew et al. (2018). It is called ATLAS (Anatomical Tracings of Lesions After Stroke) and contains 304 T1-weighted MRIs with manually segmented lesions and metadata. It may be useful to train and test stroke lesion segmentation algorithms in machine-learning. A pioneering work on a population stroke atlas was done by Bilello et al. (2006). The developed atlas contains the spatial distribution of acute infarcts in the human brain. It was created from 22 subjects with manually segmented infarcts that were subsequently registered to a common coordinate system. Another population stroke atlas was developed by Wang et al. (2019). This atlas was created from a much larger population of 793 infarct lesions of 458 patients. The infarcts were segmented and co-registered to a standard brain space. In addition, the infarcts were clustered hierarchically. It should be noted that these two approaches to creation of a population stroke atlas use ROIs type III and are simple instances of the PSA as a stroke (infarct) frequency map with averaging as an aggregation strategy. All the above stroke atlases are examples of research applications. Payabvash et al. (2010) carried out an atlas-assisted analysis of 58 consecutive stroke patients with aphasia due to first-time ischemic stroke. The authors modified the Talairach and Tournoux (1988) brain atlas in order to subdivide large subcortical areas into sub-regions for a more precise evaluation of different brain areas.

Here, we have considered four various, clinically-relevant situations concerning stroke occurrence prediction, diagnosis in the ER, decision support in thrombolysis and outcome prediction, and presented the preliminary solutions in a form of developed stroke prototypes. It should be noted that the algorithms devised to support the atlas use along with the systems developed as proof-of-concepts serve as an illustration of atlas potential. Below we discuss this preliminary work in terms of advantages, limitations, continuous enhancement, as well as potential extensions and future directions.

***Potential advantages***. The use of brain atlases in stroke has many potential advantages, such as an automated and robust handling, speed of processing, wide range of applications, and quantitative assessment.

Atlas-related operations are automated, including atlas to scan mapping and atlas-assisted analysis. These operations are also very rapid making them suitable to handle time-sensitive situations, such as stroke. For instance, the atlas to scan mapping by using the FTT takes less than 5 s (Nowinski et al. 2006b) and by employing the ellipse-based method able to handle sparse and multi-modals scans less than 15 s (Volkau et al. 2012). The atlases are color-coded so atlas-assisted analysis is fast, primary limited to traversing and comparing regions of a similar color. The PSA-based prediction requires only reading of values within a given contour from a selected PSA map (or maps). The range of atlas applications is broad and covers here prediction, diagnosis and treatment. Though not addressed in this analysis, the brain atlas is also a useful education aid in stroke, and for this purpose we have developed the *Stroke Atlas* as a 3D interactive tool correlating cerebrovascular pathology with the underlying neuroanatomy and the resulting neurological deficits (Nowinski and Chua 2013) (this stroke atlas was also trial licensed to the World Stroke Organization).

The atlas-assisted stroke occurrence prediction approach provides a wealth of measurable information, much more that is required by the traditional qualitative Fazekas scale. This information includes the number and size of WMH lesions, their spatial relationships with respect to the ventricular system, as well as their locations and contribution to the underlying anatomy including white matter tracts. To make this information clinically useful, it has to be linked to some quantitative scale. We see at least three avenues of development. First is to make the Fazekas scale quantitative by taking into account the number and size of WMH lesions. Second is to increase the granularity of the Fazekas scale, for instance, by introducing 10 quantitative grades. And third is to propose a new scale, for instance, by correlating locations and size of WMH lesions with the underlying white matter tracts obtained from the atlas. Any avenue taken, from the first, the easiest, to the last, most advanced, require much more effort. At this stage, only a preliminary enabling tool is available opening new possibilities.

The system for atlas-assisted stroke diagnosis in the ER has been designed to rapidly detect stroke and distinguish type of stroke (ischemic, hemorrhagic, or hemorrhagic transformation). It also provides lesion localization and its location with respect to anatomy and blood supply territories. The application potentially facilitates interpretation of stroke images by non-neuroradiologists in the ER (which occurs frequently before being viewed by stroke neuroradiologists) and may increase their confidence of interpretation, while enhancing a triage.

The system for atlas-assisted treatment decision making in ischemic stroke is able to calculate thrombolytic conditions. It contains two atlas-related components: calculation of the infarct to the MCA territory ratio and atlas-assisted analysis. The ratio is calculated rapidly and automatically in 3D. Note that the non-atlas determination of the MCA territory in 3D is not easy as practically it can be assessed on a single or a few images only. In fact this stroke system calculates also the other two ratios: the infarct to the territories of the anterior cerebral and posterior cerebral arteries. These two other ratios have not been applied in clinical practice yet.

The atlases make a critical difference enabling a localization analysis of infarct and penumbra, which at present is not a part of the standard procedure. The closest approach in the current state-of-the-art is the ASPECTS (Alberta Stroke Program early CT Score) aiming to standardize the detection and reporting of ischemic stroke by visually identifying an ischemic hypodensity on the MCA territory divided into ten regions on two axial CT slices (Barber et al. 2000). Our approach is conceptually superior because it quantifies both the infarct and penumbra on MR or CT by means of numerous atlas-derived regions in 3D, meaning that on all the slices or maps. Its another advantage is a possibility of a quick assessment of what functions are already lost and what are at risk by employing that version of the anatomy atlas which provides a functional description of structures (Nowinski and Thirunavuukarasuu 2004).

The main advantage of the PSA is probably its generality and flexibility. The PSA, or largely the PBDA whose construction by data aggregation mimics human experience, is a novel concept with a wide scope of potential applications. The prediction accuracy of the PSA increases with the number of cases used to construct it (Nowinski et al. 2014a). The PSA can easily be extended with new cases to increase its power and this extension can be done simply and rapidly in an incremental way provided that the averaging is used for data aggregation. However, for other aggregation approaches, the complete PSA has to be recalculated from all the previous and new cases. Another way to increase the power of the PSA is to employ a dedicated, limited and constrained PSA instance by selecting relevant parameters, determining their values or ranges, and restricting the dataset used for the PSA construction only to the cases fulfilling certain requirements. For instance, suitable limiting parameters of a patient to be predicted can be gender, age and its range, infarct volume and its range, and a stroke score at admission and its range. Then, the higher the number of limiting parameters, the lower the average prediction error (Nowinski et al. 2014a). Additionally, the employment of the anatomic atlas along with the PSA is advantageous, facilitates localization of the infarct on the PSA maps, and enables anatomic atlas-assisted analysis. A usefulness of aggregation of anatomic and neurologic information for prediction was also demonstrated by Payabvash et al. (2010) who developed a multivariate logistic regression model that could estimate the probability of early improvement in aphasia and predict functional outcome. This prediction model employs four variables: CBF of the angular gyrus and the lower third of the insular ribbon, proximal cerebral artery occlusion on admission, and aphasia score on the admission NIHSS examination.

***Limitations***. This preliminary work in progress has several limitations in terms of employed atlases, devised methods, developed stroke applications, and validation. The limitations along with their proposed overcoming regarding atlases, methods, and applications are addressed below in sections the Continuous enhancement, and Extensions and future work.

In general, validation is difficult, if possible at all in a research laboratory setting. It is a complicated, costly, tedious, and time-consuming process which additionally requires massive, heterogeneous and usually expensive data. Validation can be performed at least at three levels: research validation, clinical validation, and regulatory (FDA) validation. For instance, the cost of regulatory validation of brain-related devices is enormous, in a range of $100–200 million (BRAIN Working Group 2014).

The developed stroke systems are merely proofs of concept rather than clinically validated CAD (computer-aided diagnosis/detection/decision) systems. Consequently, a system validation and performance measurement in clinical setting is not available despite the fact that some of these stroke systems have been installed in multiple centers worldwide, yet with no reports about their clinical performance.

The methods and component algorithms employed in these stroke applications have been validated and their performance measured with numerous diverse clinical data, including the calculation of the midsagittal plane, calculation of point landmarks, fast atlas-to-scan mapping, and extraction of the ventricular system. Quite frequently these validations resulted in new, enhanced solutions (as discussed in the Continuous enhancement section).

In order to generate gold standards (ground truths) for algorithm validation, an accurate contouring of infarcts, penumbras and structures (like the cerebral ventricles) as well as the setting of point and distributed landmarks on numerous, variable scans and maps are required. Gold standard setting is time-consuming, demands attention to details, and involves the use of dedicated tools which we designed and developed. Besides the development of the dedicated validation tools, we have employed a contour editor (developed originally for atlas creation) enabling raters to accurately generate zoomed contours on axial, coronal and sagittal planes, and correlate them with the corresponding 3D polygonal models (Nowinski et al. 2012a). For instance, the FTT was validated on 215 MR datasets with a variable 0.8–3.5 mm slice thickness, requiring for each scan to set the ground truth for the midsagittal plane as a sequence of midsagittal lines on individual slices, anterior and posterior commissures and six cortical landmarks, all these resulting in the manually specifying of thousands of midsagittal lines and 1720 points in 3D (Nowinski et al. 2006b).

Some validations required a time consuming and tedious acquisition, selection and checking of suitable data. For instance, in order to build the initial version of the PSA, the data of more than 700 stroke patients were analyzed, however, to create the actual atlas only 128 of them were selected and used with a complete set of 170 parameters with one-year follow up and without other pathologies such as WMHs or hemorrhagic transformations.

Based on our experience, I believe that the problem of a successful completing of the validation of the presented stroke solutions is strictly linked to that of “crossing the chasm”, as research funding is usually limited. We tried to cross three times. In one case, a group of venture capitalists experienced in medical market from the West Coast was eager to acquire our stroke technology and start a company. However, there was a disagreement between them and my funding agency about the value of technology which blocked the conclusion of the deal. In another case, a venture capitalist from the East Coast signed a contract with my funding agency and formed a stroke company in the US. However, he was unable to secure funding from other investors to do clinical trials. In both cases, major US medical universities/hospitals were involved as clinical research partners. In addition, we negotiated with one of the companies that trial licensed one of our stroke prototypes, and the company was interested to acquire it provided that we do an FDA clearance first.

***Continuous enhancement***. This work has been continuously enhanced in terms of concepts, methods, component algorithms, and brain atlases.

We have developed several methods for mapping of atlases to scans, including (Xu and Nowinski 2001; Liu et al. 2009; Nowinski et al. 2006b; Volkau et al. 2012). The initial development was guided by applications in stereotactic and functional neurosurgery (Nowinski 2009). Although we have devised non-rigid methods for mapping of the employed here atlas of anatomy to scans, such as (Xu and Nowinski 2001; Liu et al. 2009 including Patent 8), we decided to use in stroke applications very fast and conceptually simple methods that are also potentially understandable to clinicians, including the FTT (Nowinski et al. 2006b) and the ellipse-based fitting method (Volkau et al. 2012). The FTT requires 5 s only but it was devised for MR scans. Therefore, the FTT was supplemented with the ellipse-based fitting method, although performing three times longer but being modality independent.

The component algorithms employed in the stroke applications also have been continuously enhanced, including the calculation of the midsagittal plane, calculation of landmarks for atlas-to-scan mapping, and extraction of the ventricular system. Multiple versions of the critical component algorithms have been developed to enable their performance across multiple modalities and pulse sequences as well as to handle wide and typical ranges of parameters.

The calculation of the midsagittal plane is often the first step in stroke image handling whose accuracy is critical, especially in the left to right comparison for pathology detection and for atlas-to-scan registration. We devised four algorithms for a fast, robust and accurate calculation of the midsagittal plane to accommodate to various modalities, pulse sequences and parameter ranges, namely, for MRI (Hu and Nowinski 2003), morphologic and non-morphologic images (Volkau et al. 2006a), DWI and perfusion maps (Nowinski et al. 2006c), and CT with a large head tilt (Puspitasari et al. 2009). The development of new algorithms was often driven by validation on heterogeneous, clinical data. For instance, as unenhanced CT scans in the ER have often large head tilts, so a suitable algorithm for the MSP extraction handling a large head tilt and enabling comparison of the left and right hemisphere structures was developed (Puspitasari et al. 2009). Similarly the FTT, developed originally for neurosurgery planning (Nowinski 2009) with high-resolution MR scans where the AC and PC were clearly discernible, was not suitable for scans with large slice thickness and, consequently, missing commissures. Hence, a new method based on an ellipse fitting was devised estimating these landmark locations statistically (Volkau et al. 2012).

Three algorithms were developed for the extraction of the ventricular system from MR scans (Xia et al. 2004), CT scans (Liu et al. 2010), and ischemic stroke CT scans (Poh et al. 2012). Making an algorithm anatomy and modality-dependent usually causes it to be fast (e.g., below 6 s for MR versus 10 s for CT versus 30 s for ischemic stroke CT) but often unsuitable for new applications. Moreover, a better performance for some specific task is usually at the expense of a longer execution time (in this case 3 times longer for ischemic stroke CT).

Our brain atlases have been also continuously enhanced in terms of content, quality, and creation of new atlases as addressed in (Nowinski 2005; Nowinski 2017a).

***Extensions and future work***. Future work aims to cope with the limitations in the current four stroke applications and to explore potential new atlas-assisted applications.

The proposed atlas-assisted stroke occurrence prediction approach requires more efforts in terms of definition of a new scale, atlases, and validation. As this stroke system provides a wealth of measurable information, it has to be associated to a new, manageable, quantitative, objective, more effective and useful clinical scale. The new scale has to be linked with the outcomes and its prediction performance assessed. A quantitative comparison of this new scale and the Fazekas scale shall be done. As the anatomic atlas employed so far has limited white matter tracts, a more detailed atlas of brain connections shall be employed to make the atlas-assisted analysis more specific, such as a 2D atlas by Mori et al. (2005) or a 3D atlas by Nowinski et al. (2012b). Moreover, the use of probabilistic connectional atlases, such as (Yeh et al. 2018), shall be considered. After completing this atlas extension and a performance evaluation of the new scale, a large population trial shall be performed to quantitatively link this new scale with the predicted outcomes, before this stroke system could potentially be applied prospectively for stroke occurrence screening.

Although it has been illustrated that the system for atlas-assisted stroke diagnosis in ER is able to rapidly detect stroke and distinguish type of stroke, its performance is missing. Therefore, the system sensitivity and specificity shall be determined. The detection accuracy of the atlas-assisted analysis could potentially be increased by combining it with the stroke imaging marker (SIM) whose overall detection accuracy was 83% examined on 576 stroke patients with unenhanced CT (Nowinski et al. 2013). Then the SIM calculation, performed originally for the complete hemisphere, could be restricted to blood supply and other territories making it more sensitive and specific. Another advantage of the SIM in the ER is that the SIM-based stroke detection also substantially outperformed novice readers and was able to detect early infarcts (with the time after the onset of symptoms at acquisition not longer than 3 h) that were missed by stroke neuroradiologists (Nowinski et al. 2013).

The system for atlas-assisted treatment decision making in ischemic stroke is probably the most novel. However, despite being awarded at several clinical meetings, and most frequently trial licensed to companies such as Philips and Siemens and numerous hospitals worldwide, its clinical validation has not been completed. Its performance shall be compared with that of the ASPECTS scale, which may be a turning point in its clinical acceptance. Moreover, a finer parcellation of the atlas of blood supply territories could improve an atlas-assisted analysis. An automatic delineation of the penumbra shall also be included. Finally, more advanced brain atlases shall be employed in terms of population, specimen age range span, and age appropriateness, such as the atlas developed by Liang et al. (2015) that contains a large number of 2020 specimens whose age spans from 20 to 75 years at 5 year interval.

In order to make the PSA useful clinically, and in particular to be able to generate dedicated PSA versions with a suitable statistical power, probably tens of thousands of cases shall be employed (in contrast to the current 128 cases selected from more than 700 patients). To test the performance of the PSA, about 75 millions of combinations resulting from the current number of cases as well as various situations and ROI aggregation approaches were analyzed (Nowinski et al. 2014a). This number would escalate dramatically should the number of cases be drastically increased.

Similarly, the PSA can also be created for hemorrhagic stroke, although the scan normalization will be more demanding because of substantial anatomical distortions caused by hemorrhages of variable size, shape and location (then, for instance, a fast method of atlas-to-scan registration for brain tumors causing mass effect could be attempted (Nowinski and Belov 2005)). A suitable environment for the creation of the hemorrhagic PSA could be the hemorrhagic stroke CAD (Nowinski et al. 2014b) which provides clot segmentation, quantification, and visualization. The system has been developed to handle intraventricular hemorrhagic stroke cases from the CLEAR III Interventricular Hemorrhage Clinical Trial [URL, CLEAR 2020] and intracerebral hemorrhagic stroke cases from the MISTIE III Clinical Trial (Minimally Invasive Surgery Plus rt-PA for Intracerebral Hemorrhage Evacuation) [URL, MISTIE 2020]. The CLEAR and MISTIE are international stroke phase III multicenter clinical trials with multi-million funding that acquire imaging data and functional outcomes defined with the modified Rankin Scale. The wealth of data, including scans, segmented hemorrhages and long term functional outcomes, acquired by the MISTIE and CLEAR potentially enable the construction of a hemorrhagic PSA. Then, the hemorrhagic CAD could also be extended with PSA-based outcome prediction capabilities.

A natural extension of this work is a combination of these four stroke applications into a single stroke suite, a sort of pipeline for stroke occurrence screening, detection, treatment, and outcome prediction. Moreover, the atlas-based approaches can be combined with other methods, such as artificial intelligence besides advanced image processing and analysis.

***In summary***, we have presented novel concepts and methods in stroke management by employing multiple human brain atlases applied in a new way. We have also addressed a potential usefulness of and summarized our experience in using various brain atlases for stroke prediction, diagnosis and treatment.

The method employs three brain atlases: atlas of anatomy, atlas of blood supply territories, and probabilistic stroke atlas (which is a special case of a probabilistic brain damage atlas). It rapidly superimposes an atlas to a scan or perfusion map and provides atlas-assisted scan processing. An atlas-to-scan mapping is application-dependent and handles certain regions of interest (ROIs) in the scan or map. An ROI can be defined either by atlas-guided anatomy (including blood supply territories) or scan-derived pathology. The atlas can be applied either to define an ROI or to quantify it. Three situations of an ROI employment have been considered and, consequently, three types of ROIs distinguished: atlas-defined ROIs (type I), atlas-quantified ROIs (type II), and ROIs creating an atlas (type III).

An atlas potential has been illustrated in four atlas-assisted applications for stroke occurrence prediction and screening (based on type II ROIs), rapid and automatic stroke diagnosis in the ER (based on type I ROIs), quantitative decision support in thrombolysis in ischemic stroke (based on type II ROIs), and stroke outcome prediction and treatment assessment (with type III ROIs employed for atlas creation and type II ROIs used in prediction).

The application of brain atlases in stroke has many potential advantages, including an automated and robust handling, speed of processing, wide range of applications, and quantitative assessment.

Future directions are determined in terms of atlases, methods, validation, and stroke applications. Further extensive work is needed to enhance the developed methods and prototypes, clinically validate the proposed solutions, and to introduce them to clinical practice.

### Information Sharing Statement

The presented stroke prototypes belong to their funding organization, Agency for Science, Technology and Research (A*STAR), Singapore. They may be obtained from Exploit Technologies, the technology transfer office of A*STAR, by contacting techoffer@exploit-tech.com.

## Appendix: Related US patents granted in neuroimage processing, brain atlases and stroke (out of total 71 patents granted and 68 pending)

1. Bhanu Prakash KN, Gupta V, Nowinski WL: *Segmenting infarct in diffusion-weighted imaging volumes.* US Patent No.: US 8125223 B2, Date of Patent: Feb. 28, 2012. https://patents.google.com/patent/US8125223B2
2. Bhanu Prakash K N, Volkov I, Nowinski WL: *Locating a mid-sagittal plane*. US Patent No.: US 7822,456 B2, Date of Patent: Oct. 26, 2010. http://www.freepatentsonline.com/7822456.html
3. Bhanu Prakash K N, Nowinski WL: *Automatic identification of the anterior and posterior commissure landmarks*. US Patent No. US7,783,090 B2 granted on 24 Aug. 2010; www.freepatentsonline.com/7783090.html
4. Gupta V, Nowinski WL: *Method and system for segmenting a brain image.* US Patent No.: US 8,831,328 B2, Date of Patent: Sep. 9, 2014. https://patents.google.com/patent/US8831328B2
5. Gupta V, Bhanu Prakash KN, Nowinski WL: *Method for identifying a pathological region of a scan, such as an ischemic stroke region of an MRI scan*. US Patent No.: US 8,369,598 B2, Date of Patent: Feb 5, 2013. https://patents.google.com/patent/US8369598B2
6. Hu Q, Nowinski WL: *Automated method for identifying landmarks within an image of the brain*. US Patent No. US 7,715,607 B2 granted on 11 May 2010; www.freepatentsonline.com/7715607.html
7. Hu Q, Nowinski WL: *Method and apparatus for determining symmetry in 2D and 3D images*. US Patent No.: 7,409,085 B2, Date of Patent: 5 August 2008. https://patents.google.com/patent/US7409085B2
8. Liu JM, Huang S, Nowinski WL: *Method and apparatus for registration of an atlas to an image*. US Patent No.: US 8,687,917 B2, Date of Patent: Apr. 1, 2014. https://patents.google.com/patent/US8687917B2
9. Nowinski WL: *Detection and localization of vascular occlusion from angiography data*. US patent no. US 8,050,475 granted on 1 Nov 2011. https://patents.google.com/patent/US8050475B2
10. Nowinski WL, Beauchamp N: S*uperimposing brain atlas images and brain images with delineation of infarct and penumbra for stroke diagnosis*. US patent No.: US 8,019,142 B2, Date of Patent: Sep 13, 2011. https://patents.google.com/patent/US8019142B2
11. Nowinski WL, Hu Q: *Method and apparatus for identifying pathology in brain images*. US Patent No.: US 7,889,895 B2, Date of Patent: Feb. 15, 2011. https://patents.google.com/patent/US7889895B2
12. Nowinski WL, Thirunavuukarasuu A: *Method and apparatus for processing medical images*. US patent No. 7646898 B1, Date of Patent: 12 Jan 2010. https://patents.google.com/patent/US7646898B1
13. Nowinski WL, Bhanu Prakash KN, Volkau I, Ananthasubramaniam A: *Method and apparatus for atlas-assisted interpretation of magnetic resonance diffusion and perfusion images*. US patent No.: US 7,783,132 B2, Date of Patent: 24 August 2010. https://patents.google.com/patent/US7783132B2
14. Nowinski WL, Xia Y, Aziz A, Hu Q: *Method and apparatus for extracting cerebral ventricular system from images*. US Patent No.: US 7,756,306 B2, Date of Patent: Jul. 13, 2010. http://www.freepatentsonline.com/7756306.html
15. Volkau I, Nowinski WL: *Method and apparatus for determining asymmetry in an image*. US Patent No.: U S7,805,001 B2, Date of Patent: 28 Sep 2010. https://patents.google.com/patent/US7805001B2
16. Volkau I, Bhanu Prakash KN, Ng TT, Gupta V, Nowinski WL: *Localization of brain landmarks such as the anterior and posterior commissures based on geometrical fitting*. US Patent No.: US8,045,775 B2, Date of Patent: 25 Oct 2011. https://patents.google.com/patent/US8045775B2
17. Volkau I, Bhanu Prakash KN, Ng TT, Gupta V, Nowinski WL: *Registering brain images by aligning reference ellipses.* US Patent No.: US 8,311,359 B2, Date of Patent: Nov. 13, 2012. https://patents.google.com/patent/US8311359B2
